# Sigma1 Receptor Activation Confers Durable Neuroprotection Following Neonatal Ischemic Retinal Injury

**DOI:** 10.21203/rs.3.rs-10029409/v1

**Published:** 2026-06-23

**Authors:** Jing Wang, Xiaowen Lu, Zhengyu Lu, Zhimin Xu, Sylvia B. Smith, Steven E. Brooks, Ruth B. Caldwell

**Affiliations:** 1. Department of Cellular Biology and Anatomy, Medical College of Georgia at Augusta University, Augusta, GA 30912, USA.; 2. James and Jean Culver Vision Discovery Institute, Augusta University, Augusta, GA 30912, USA.; 3. Vascular Biology Center, Augusta University, Augusta, GA 30912, USA.; 4. Department of Ophthalmology, Medical College of Georgia at Augusta University, Augusta, GA 30912.

**Keywords:** Retinopathy of prematurity, oxygen-induced retinopathy, Sigma 1 receptor, neuroprotection, oxidative stress, mitochondrial dysfunction, retinal neurodegeneration

## Abstract

Retinopathy of prematurity (ROP) remains a leading cause of childhood blindness. Although current therapies effectively suppress pathological neovascularization, many patients continue to exhibit persistent visual dysfunction despite regression of active disease, highlighting an unmet need for neuroprotective interventions. Sigma 1 receptor (Sig1R), an endoplasmic reticulum-mitochondrial chaperone and regulator of cellular stress responses, has emerged as a promising therapeutic target in neurodegenerative and retinal diseases. Here, we investigated whether Sig1R activation confers sustained neuroprotection following neonatal ischemic retinal injury. Wild-type and Sig1R knockout mice were subjected to oxygen-induced retinopathy (OIR) and treated systemically with the high-affinity Sig1R agonist (+)-pentazocine [(+)-PTZ]. Retinal structure and visual function were assessed longitudinally through 20 weeks of age using visual acuity, contrast sensitivity, electroretinography (ERG), pattern ERG (PERG), spectral-domain optical coherence tomography (SD-OCT), and histological analyses. Chronic Sig1R activation significantly preserved visual acuity, contrast sensitivity, rod- and ganglion cell-mediated retinal function, retinal ganglion cell survival, and inner retinal architecture in OIR mice. These protective effects were abolished in Sig1R-deficient mice, demonstrating a requirement for Sig1R in mediating neuroprotection. Mechanistically, Sig1R activation reduced apoptotic signaling, attenuated oxidative and nitrosative stress, improved mitochondrial respiratory function, and enhanced endogenous antioxidant pathways. Collectively, these findings demonstrate that Sig1R activation provides durable, receptor-dependent neuroprotection following neonatal ischemic retinal injury by coordinating redox, mitochondrial, and cell-survival pathways. These results identify Sig1R as a promising therapeutic target for preserving retinal neuronal integrity and long-term visual function in retinopathy of prematurity.

## 1. Introduction

Retinopathy of prematurity (ROP) is a leading cause of childhood blindness and remains a major unmet clinical challenge (Blazon et al. 2024). In the United States alone, 14,000–16,000 infants develop ROP annually, with 400–600 progressing to legal blindness, and the incidence continues to rise as survival of extremely preterm infants improves. Although clinically classified as a retinal vascular disorder characterized by ischemia-driven neovascularization (NV) and in severe cases tractional retinal detachment, current standards of care, including laser photocoagulation and intravitreal anti-VEGF, primarily suppress pathological NV without restoring normal retinal development or preventing long-term visual dysfunction (Nishijima et al. 2007; Lim et al. 2011; Higashide et al. 2012).

Increasing clinical and experimental evidence indicates that ROP is a neurovascular disease of the developing retina, rather than a purely vascular disorder (Fletcher et al. 2010; Hansen et al. 2017). Despite anatomically successful treatment, many children exhibit persistent deficits in visual acuity, contrast sensitivity, and electrophysiolgical responses, including delayed ERG responses (Hansen et al. 2017; Fulton et al. 2009). rod pathway dysfunction and photoreceptor deficits can persist for years after regression of neovascularization, indicating sustained injury to the neural retina rather than isolated pathology (Fulton and Hansen 1996; Fulton et al. 2001; O’Connor et al. 2002; Yassin et al. 2019). Together, these findings highlight dysfunction of the retinal neurovascular unit as a central but under-targeted driver of long-term visual impairment in ROP.

A growing body of evidence implicates shared cellular stress pathways, including oxidative stress, mitochondrial dysfunction, and apoptosis, as key mediators of neurovascular injury in ROP. Premature infants are exposed to repeated oxygen fluctuations during a critical window of retinal development, rendering both neuronal and vascular components of the neurovascular unit highly vulnerable to redox imbalance and bioenergetic failure (Fulton and Hansen 1996; Fulton et al. 2009; Fulton et al. 2001; Fletcher et al. 2010; Narayanan et al. 2011). These convergent stress mechanisms suggest that therapeutic strategies aimed at restoring cellular homeostasis may be more effective than angiogenesis inhibition alone.

Sigma 1 receptor (Sig1R) is a unique endoplasmic reticulum (ER) chaperon that functions as a central regulator of cellular stress signaling (Schmidt et al. 2016). Sig1R modulates Ca^2+^ homeostasis (Hayashi and Su 2003) and regulates stress-responsive transcriptional pathways, including NFκB (Meunier and Hayashi 2010; Shanmugam et al. 2015) and Nrf2 signaling (Xiong et al. 2015; Wang, Saul, et al. 2016). Importantly, Sig1R has emerged as a key modulator of mitochondrial function, oxidative stress, and cell survival across both central nervous system (CNS) and retinal disease models.

The high-affinity Sig1R ligand (+)-pentazocine [(+)-PTZ] (Fontanilla et al. 2009; Walker et al. 1990) has demonstrated broad neuroprotective effects in CNS (Hayashi and Su 2007; Su et al. 2016; Hayashi 2019; Meunier and Hayashi 2010), and retinal degenerative models, including preservation of retinal ganglion cells (RGCs) (Zhao et al. 2016; Wang, Cui, et al. 2016; Martin et al. 2004; Dun et al. 2007; Cantarella et al. 2007; Smith et al. 2008) and photoreceptors (Wang, Saul, et al. 2016; Wang et al. 2019; Wang et al. 2023; Wang et al. 2021; Wang et al. 2020), as well as suppression of oxidative and ER stress (Ha et al. 2011; Wang et al. 2015; Mueller et al. 2013; Ha et al. 2014; Tchedre et al. 2008). Beyond neuroprotection, emerging evidence from our group indicates that Sig1R also attenuates vascular injury in oxygen-induced retinopathy (OIR), suggesting a broader role in regulating ischemic responses within the neurovascular unit (Wang 2026). However, whether Sig1R activation can preserve long-term neurovascular unit function and visual outcomes in ROP through integrated regulation of neuronal and metabolic stress pathways remains unknown.

In this study, we investigated whether pharmacological activation of Sig1R provides sustained neuronal and functional protection in OIR mouse model of ROP. We demonstrate that long-term (+)-PTZ treatment preserves visual function, retinal structure, and neuronal survival. Mechanistically, Sig1R activation suppresses oxidative and nitrosative stress, enhances mitochondrial respiratory function, activates Nrf2-dependent antioxidant signaling, and reduces intrinsic apoptotic pathways. Importantly, these protective effects are abolished in Sig1R-deficient mice, confirming Sig1R dependence. Together, our findings identify Sig1R as a central regulator of neurovascular unit homeostasis in ischemic retinopathy and support Sig1R activation as a potential disease-modifying strategy for ROP, as well as retinal and CNS neurovascular diseases characterized by hypoxic and oxidative stress.

## Results:

### 2.1. Sig1R activation improves visual acuity and contrast sensitivity in OIR mice.

**Fig.1:**
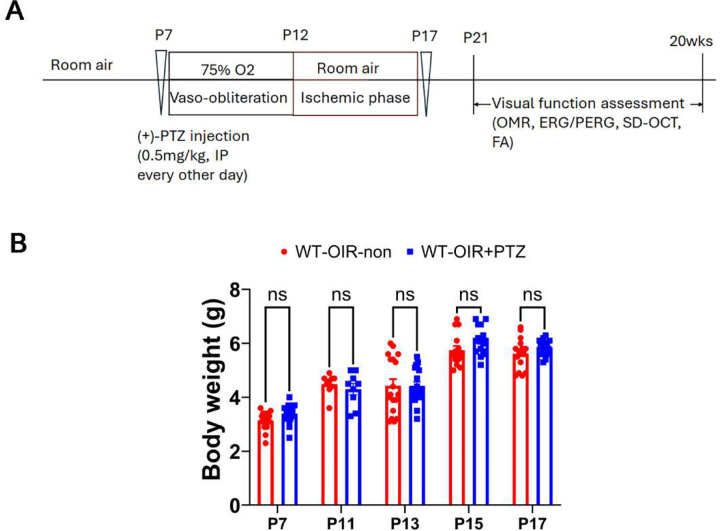
(A) Experimental design and treatment timeline. (B) Body weight curve with or without (+)-PTZ in WT-OIR mice from P7 to P17.

The experimental design is illustrated in Figure 1A. While the (+)-PTZ treatment did not show any detrimental effects on animal phenotype, including body weight (Fig.1B), our longitudinal study demonstrated that Sig1R activation provided long-term neuroprotection, with visual assessments performed from P21 (3 weeks) through 20 weeks of age.

**Fig.2:**
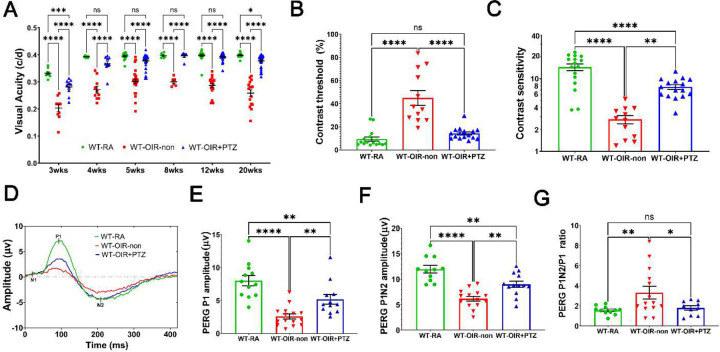
Sig1R activation preserves visual function in OIR mice. (**A)** Visual acuity at 3, 4, 5, 8, 12, 20 weeks. (**B)** Contrast threshold at 5 wks. (**C)** Contrast sensitivity. (**D)** Averaged PERG traces from WT-RA, WT-OIR-non and WT-OIR+PTZ mice. (**E)** PERG P1 amplitude. (**F)** PERG P1N2 amplitude. (**G)** PERG P1N2/P1 ratio. Values are mean ± S.E.M. (n=8–15 mice per group). *, *p* < 0.05; ***, *p* < 0.001, ****, *p* < 0.0001. WT-RA: Wild-type mice room air control; WT-OIR-non: wild-type OIR non-treated group; WT-OIR+PTZ: wild-type (+)-PTZ treated-OIR group.

First, OptoMotry (OMR) test indicated that (+)-PTZ significantly improved visual acuity and contrast sensitivity in OIR mice. At 3 weeks, visual acuity (c/d) in untreated OIR wild type mice (WT-OIR-non) was markedly reduced (WT-OIR-non: 0.20 ± 0.02) compared with wild-type room air controls (WT-RA: 0.33 ± 0.02), whereas (+)-PTZ-treated OIR wild type mice (WT-OIR+PTZ) exhibited significant improvement (WT-OIR+PTZ: 0.28 ± 0.03) (Fig.2A). From 4–20 weeks, visual acuity of WT-RA mice remained at ~0.40 c/d, while WT-OIR-non mice maintained reduced visual acuity (0.26–0.30 c/d). In contrast, WT-OIR+PTZ mice demonstrated improved visual acuity at 0.37–0.4 c/d, comparable to WT-RA levels from weeks 4 to 12 (Fig.2A).

Contrast threshold (%) was significantly elevated in WT-OIR-non mice compared to WT-RA mice (WT-OIR-non: 45.03 ± 22.21 vs. WT-RA: 9.48 ± 7.18). In contrast, (+)-PTZ treatment reduced the contrast threshold in WT-OIR+PTZ mice (WT-OIR+PTZ: 14.30 ± 5.18) to WT-RA levels (Fig.2B). On the other hand, WT-OIR-non mice showed a marked reduced contrast sensitivity compared with WT-RA (WT-WOIR-non: 2.75 ± 1.27 vs. WT-RA: 14.50 ± 6.26). However, WT-OIR+PTZ mice exhibited a significant improvement in contrast sensitivity (WT-OIR+PTZ: 7.72 ± 2.34) (Fig.2C).

### 2.2. Sig1R activation preserves long-term retinal function in OIR

Given that contrast sensitivity reflects retinal ganglion cell (RGC) function, we then performed pattern electroretinogram (PERG) to further evaluate RGC function (Fig. 2 D–G). WT-OIR-non mice exhibited a flattened PERG averaged trace and reduced P1 and P1N2 amplitudes compared to WT-RA mice. However, (+)-PTZ treatment restored PERG trace (Fig.2D) and significantly increased the amplitudes of both P1 (WT-RA: 8.00 ± 2.72 μV; WT-OIR-non: 2.59 ± 1.49 μV; WT-OIR+PTZ: 5.19 ± 2.52 μV) (Fig. 2E) and P1N2 (WT-RA: 11.95 ± 2.44 μV; WT-OIR-non: 6.16 ± 1.80 μV; WT-OIR+PTZ: 8.99 ± 2.21 μV) (Fig.2F). Additionally, the elevated P1N2/P1 ratio observed in WT-OIR-non mice was normalized by (+)-PTZ treatment in WT-OIR+PTZ mice, comparable to WT-RA level (WT-RA: 1.57 ± 0.42; WT-OIR-non: 3.32 ± 2.34; WT-OIR+PTZ: 1.80 ± 0.69) (Fig.2G).

**Fig.3:**
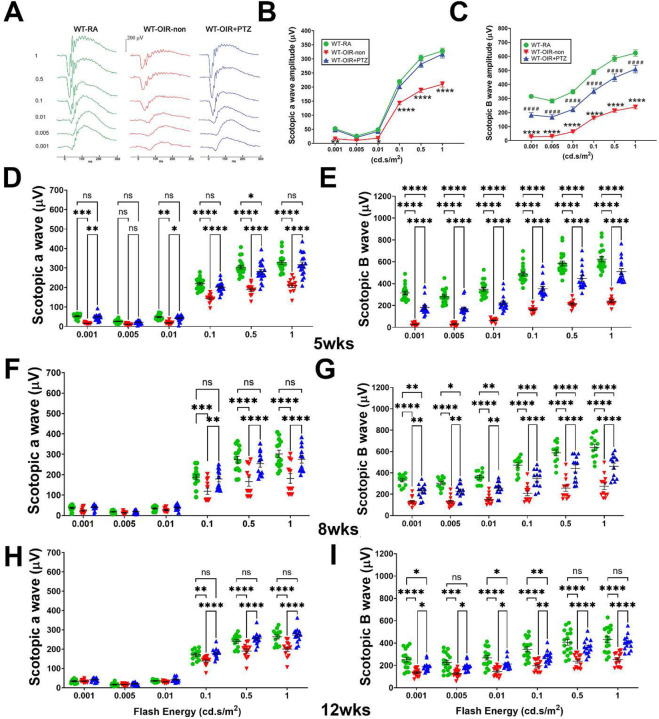
Sig1R activation sustains scotopic ERG in OIR mice. (**A)** Averaged scotopic ERG traces from WT-RA, WT-OIR-non and WT-OIR+PTZ mice at 5 wks. (**B**) Scotopic ERG a-wave amplitude. (**C)** Scotopic B-wave amplitude. **(D-I)** Long-term Scotopic ERG function test from 5, 8 to 12 wks. Scotopic a and B wave amplitudes from WT-RA, WT-OIR-non and WT-OIR+PTZ groups at 5 wks. (**D, E**), 8 wks. **(F, G)** and 12wks (**H, I**). Values are mean ± S.E.M. (n=8–15 mice per group). *, *p* < 0.05; ***, *p* < 0.001, ****, *p* < 0.0001.

Scotopic ERG was further performed to determine whether activation of Sig1R by (+)-PTZ provides long-term visual restoration via the rod to bipolar cell pathway. WT-OIR-non mice exhibited a significantly reduced a-wave, suggesting rod dysfunction (Fig.3A–C). Bipolar cell function indicated by the B-wave amplitudes in WT-OIR-non, was shown to be markedly impaired compared to WT-RA controls (Fig.3C). In contrast, WT-OIR+PTZ mice displayed robust elevated both a- and B-wave responses (Fig.3A–C). Quantitatively, the a-wave amplitudes in WT-OIR+PTZ mice were restored to normal WT-RA levels (Fig.3B), while the B-wave amplitudes recovered to approximately half of WT-RA values (Fig.3C). Longitudinal assessments at 5, 8, and 12 weeks revealed consistent robust protection by activation of Sig1R. WT-OIR+PTZ mice maintained significantly higher a- and B-wave amplitudes compared to WT-OIR-non mice at all time points (Fig.3D–I). At 5, 8 and 12 weeks of age, a-wave amplitudes of WT-OIR+PTZ mice were comparable to WT-RA levels, while most of the B-wave amplitudes maintained over 50% recovery compared to WT-RA levels (Fig.3D–I).

### 2.3. Sig1R activation prevents retinal structural degeneration in OIR mice

**Fig. 4:**
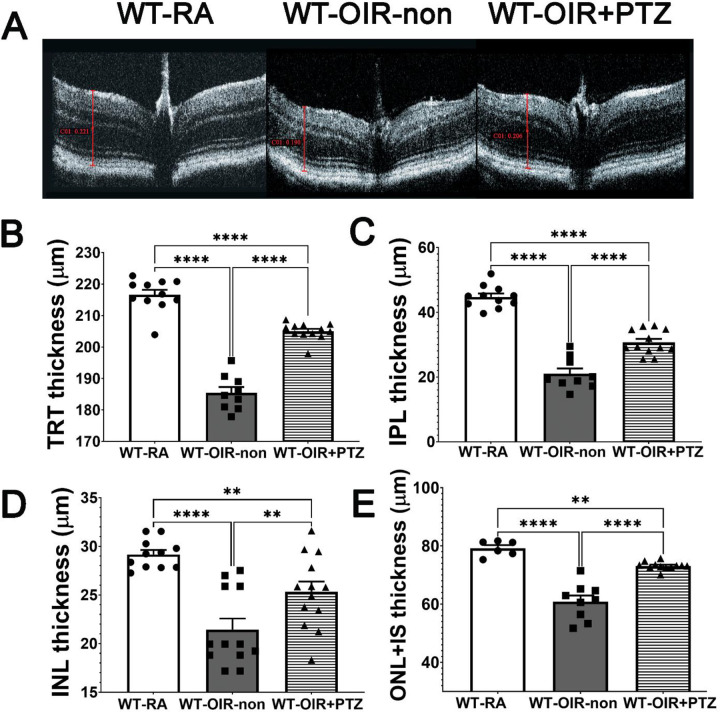
Sig1R activation preserves *in vivo* retinal structure in OIR mice demonstrated by SD-OCT. **(A)** Representative SD-OCT images from WT-RA, WT-OIR-non and WT-OIR+PTZ groups at 8wks. Quantification of SD-OCT data including total retinal thickness (TRT) (**B**), inner plexiform layer (IPL) (**C**), inner nuclear layer (INL) (**D**) and retinal nerve fiber layer (RNFL) (**E**). **, *p* < 0.01, ****, *p* < 0.0001. Data are the mean ± SEM of analyses in 5–10 mice per group.

We monitored retinal structure using spectral domain optical coherence tomograph (SD-OCT). Representative images from WT-RA, WT-OIR-non, and WT-OIR+PTZ mice at 8wks are shown in Fig. 4A. Retinal measurements revealed that (+)-PTZ treatment significantly preserves retinal thickness in OIR mice. First, the total retinal thickness (TRT) was significantly decreased in WT-OIR-non mice (185.45 ± 5.62 μm) compared to WT-RA mice (221.08 ± 3.69 μm). In contrast, (+)-PTZ significantly increased the TRT in WT-OIR+PTZ mice (205.03 ± 2.72 μm (Fig. 4B). Layer-specific analysis showed that the inner plexiform layer (IPL, Fig. 4C) (IPL: WT-OIR-non: 21.00 ± 4.88 μm vs. WT-RA: 52.59 ± 3.33 μm) and inner nuclear layer (INL, Fig. 4D) (INL: WT-OIR-non: 24.50 ± 4.57 μm vs. WT-RA: 29.17 ± 1.50 μm) were both significantly decreased in WT-OIR-non compared with WT-RA mice. However, both IPL and INL were preserved in WT-OIR+PTZ mice (IPL: WT-OIR+PTZ: 30.68 ± 3.78 μm; INL: WT-OIR+PTZ: 25.15 ± 3.82 μm). Retinal outer nuclear layer plus inner segment (ONL+IS) was also significantly decreased in WT-OIR-non mice, however markedly increased in WT-OIR+PTZ mice (ONL+IS: WT-RA: 79.20 ± 2.61; WT-OIR-non: 60.88 ± 6.23; WT-OIR+PTZ: 73.16 ± 1.33) (Fig.4E).

### 2.4. Sig1R activation preserves inner retinal neurons and synaptic layers in OIR

**Fig. 5:**
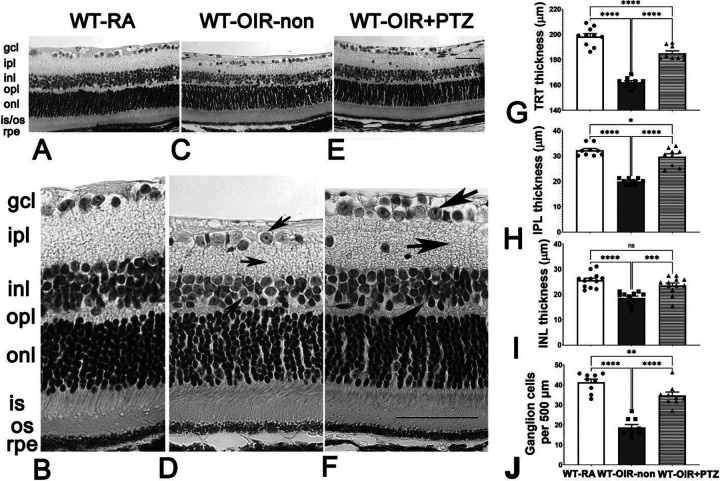
Sig1R activation protects retinal neurons in OIR mice by JB4 staining. (**A-F**) JB4 stained retinal section (**A, C, E**: lower magnification, scale bar: 50 μm; **B, D, F**: higher magnification, scale bar: 50 μm); arrows indicated ganglion cell, IPL and INL respectively. Quantification of retinal layer thickness including TRT (**G**), IPL (**H**), INL (**I**) and ganglion cells number per 500μm (**J**). *, *p* < 0.05; **, *p* < 0.01, ****, *p* < 0.0001. Data are the mean ± SEM of analyses in 5–10 mice per group.

Histological analysis showed that the outer retina in WT-OIR-non was largely comparable to WT-RA, with a mild reduction in IS that was normalized by (+)-PTZ (Fig.5A–F). In contrast, the inner retina was markedly compromised in WT-OIR-non mice, with significant thinning of the INL and IPL and reduced ganglion cell numbers (Fig.5D arrow). (+)-PTZ treatment preserved inner retinal structure, maintaining thicker INL and IPL layers and higher RGC counts (Fig.5F arrow). Morphometric analysis confirmed significant degeneration in WT-OIR-non mice vs. WT-RA, including reduced TRT thickness (WT-OIRnon: 162.11 ± 3.88 μm vs. WT-RA: 198.30±7.38 μm) (Fig.5G), IPL (WT-OIR-non: 19.97 ± 1.17 μm vs. WT-RA: 32.24 ± 2.28 μm) (Fig.5H) and INL (WT-OIR-non: 19.01 ± 2.38 μm vs. WT-RA: 26.06 ± 3.06 μm) (Fig.5I) and ganglion cells per 50 μm across the section (WT-OIR-non: 18.68 ± 4.56 vs. WT-RA: 41.34 ± 4.75) (Fig.5J). (+)-PTZ significantly improved TRT (185.18 ± 5.38 μm), IPL (29.70 ± 3.55 μm) and INL (24.59 ± 2.39 μm), RGCs counts (34.71 ± 5.27) compared to WT-OIR-non mice (Fig.5 G–J).

### 2.5. Genetic deletion of Sig1R abolishes (+)-PTZ-mediated neuroprotection in OIR mice

**Fig.6:**
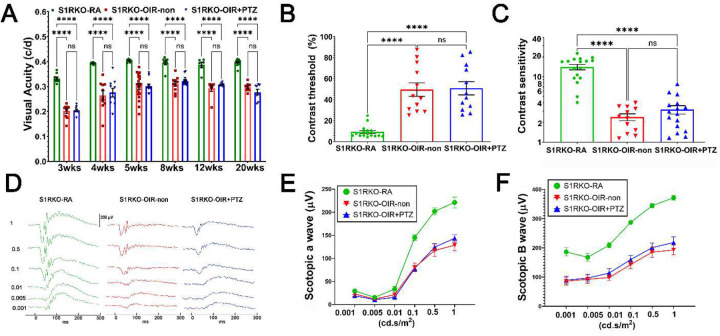
(+)-PTZ-mediated visual function protection is absent in Sig1R^−/−^ OIR mice. **(A)** Visual acuity of S1R^−/−^ (S1RKO) mice exposed to OIR model with or without (+)-PTZ administration at 3, 4, 5, 8, 12, 20 wks. (**B**) Contrast threshold and (**C**) contrast sensitivity of S1RKO-RA, S1RKO-OIR-non and S1R-OIR+PTZ mice. (**D**) Averaged scotopic ERG traces from S1RKO-RA, S1RKO-OIR-non and S1RKO-OIR+PTZ mice. (**E**) Scotopic ERG a-wave amplitude. (**F)** Scotopic B-wave amplitude at 5wks. Data are the mean ± SEM of analyses in 6 mice per group. ****, *p* < 0.0001. S1RKO-RA: Sig1R^−/−^ mice room air control; S1RKO-OIR-non: Sig1R^−/−^ mice OIR non-treated group; S1RKO-OIR+PTZ: Sig1R^−/−^ mice (+)-PTZ treated-OIR group.

**Fig.7:**
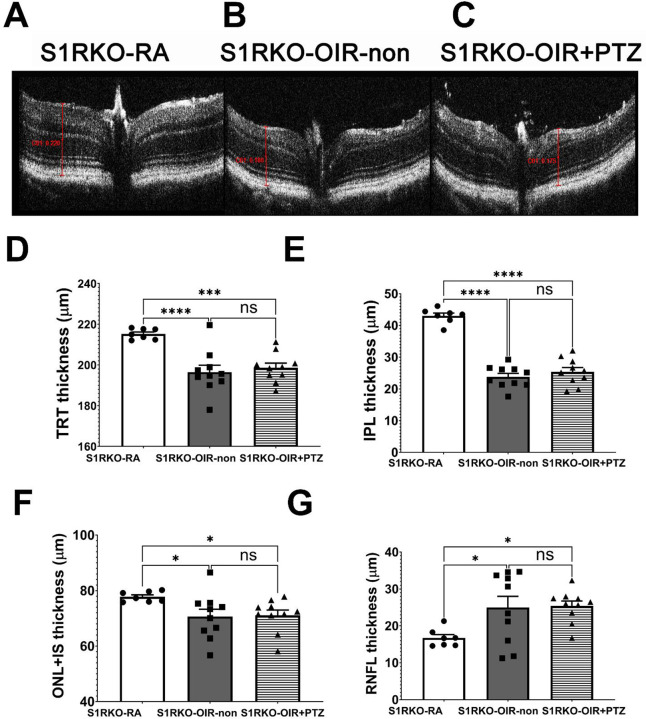
Absence of (+)-PTZ-mediated retinal structure protection in Sig1R^−/−^ OIR mice. **(A-C**) Representative SD-OCT images from S1RKO-RA (**A**), S1RKO-OIR-non (**B**) and S1RKO-OIR+PTZ (**C**) at 8wks. Quantification of SD-OCT data including total retinal thickness (TRT) (**D**), inner plexiform layer (IPL) (**E**), ONL+IS layer (**F**) and RNFL (**G**). *, *p* < 0.05; ***, *p* < 0.001, ****, *p* < 0.0001. Data are the mean ± SEM of analyses in 5–10 mice per group.

To determine whether (+)-PTZ-mediated neuroprotection is exclusively Sig1R-dependent, we induced OIR in Sig1R^−/−^ mice with or without (+)-PTZ and assessed visual function (OMR, ERG), and structure (SD-OCT). OIR significantly reduced visual acuity in Sig1R^−/−^ mice (S1RKO-OIR-non) compared with Sig1R^−/−^ room air controls (S1RKO-RA) across all ages tested (3–20wks), and (+)-PTZ failed to improve visual acuity, contrast threshold or contrast sensitivity (Fig.6A–C). ERG showed robust scotopic responses in S1RKO-RA controls but markedly diminished responses in Sig1R^−/−^ OIR regardless of (+)-PTZ treatment (Fig.6D), with reduced a-wave (Fig.6E) and B-wave amplitudes (Fig.6F) in both OIR groups. Similarly, SD-OCT (Fig.7A) and segmentation revealed no difference in TRT (Fig.7D), IPL(Fig.7E), ONL (Fig.7F), or retinal fiber layer (RNFL) thickness (Fig.7G) between treated and untreated Sig1R^−/−^ mice. Collectively, these data demonstrate that Sig1R deficiency abolishes (+)-PTZ-mediated retinal neuroprotection, indicating that Sig1R is essential for its neuroprotective effects in OIR.

### 2.6. Sig1R activation reduces retinal neuronal cell death in OIR mice

**Fig.8:**
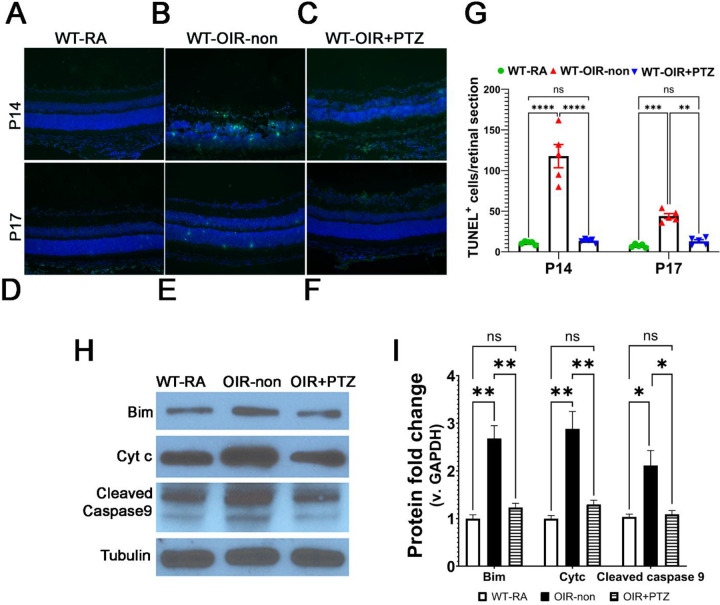
Sig1R activation attenuates retinal neuronal cell death. TUNEL assay detection of retina cryosections from WT-RA, WT-OIR-non and WT-OIR+PTZ mice. Representative TUNEL assay image of WT-RA, WT-OIR-non and WT-OIR+PTZ group at P14 (**A-C**) and P17 (**D-F**), respectively. Quantification of cell death by TUNEL assay (**G**). (H,I) Bim, cytochrome C and Cleaved caspase 9 protein level change shown by Western blotting (H) and density quantification (I). *, *p* < 0.05; **, *p* < 0.01, ***, *p* < 0.001, ****, *p* < 0.0001. Data are the mean ± SEM of analyses in 3–5 replicates.

To determine whether Sig1R activation reduce OIR-induced retinal cell death, we performed TUNEL assays at P14 (2 days post-hyperoxia) and P17 (NV peak day). OIR-non retinas showed numerous TUNEL-positive cells across the INL, ONL, and RGC layers at P14 (Fig.8B), whereas WT-OIR+PTZ retinas had minimal TUNEL labeling, comparable to WT-RA (Fig. 8C). By P17, residual TUNEL labeling in OIR-non mice was mainly in the ONL (Fig.8E), while WT-OIR+PTZ retinas remained largely free of apoptotic cells (Fig.8 F) as confirmed by quantification (Fig.8G). Mechanistically, OIR-non retinas exhibited increased Bim, cytochrome C releases, and cleaved caspase-9 indicative of mitochondrial apoptosis, all of which were markedly suppressed by (+)-PTZ (Fig.8H, I). These results indicate that Sig1R activation protects against OIR-induced neuronal injury by inhibiting mitochondria-mediated apoptotic pathway.

### 2.7. Sig1R activation suppresses oxidative and nitrosative stress in OIR retina

**Fig.9:**
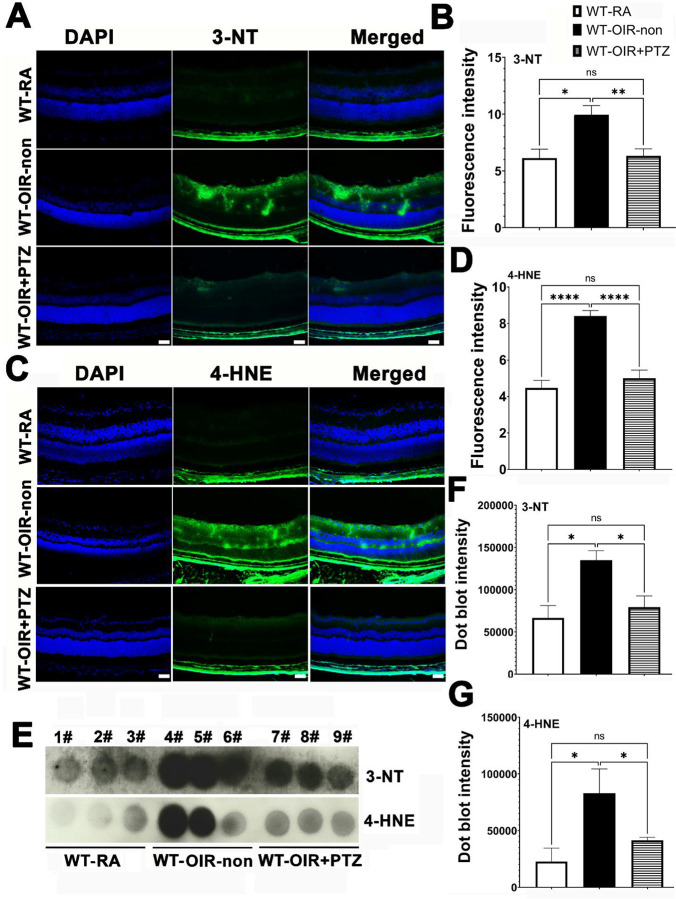
(+)-PTZ inhibits oxidative and nitrosative damage in OIR. (**A-B**) 3-NT (**C-D**) and 4-HNE staining and fluorescence intensity quantification for retinal cryosection from WT-RA, WT-OIR-non and WT-OIR+PTZ groups at P17. Dot blot assay of 3-NT and 4-HNE (**E**) and intensity quantification (**F, G**) for retinal lysates from WT-RA, WT-OIR-non and WT-OIR+PTZ mice. Scale bar: 50 μm. Values are mean ± SEM. Data are the mean ± SEM of analyses in 6 mice per group. *, p < 0.05; **, p < 0.01; ****, p < 0.0001.

**Fig.10:**
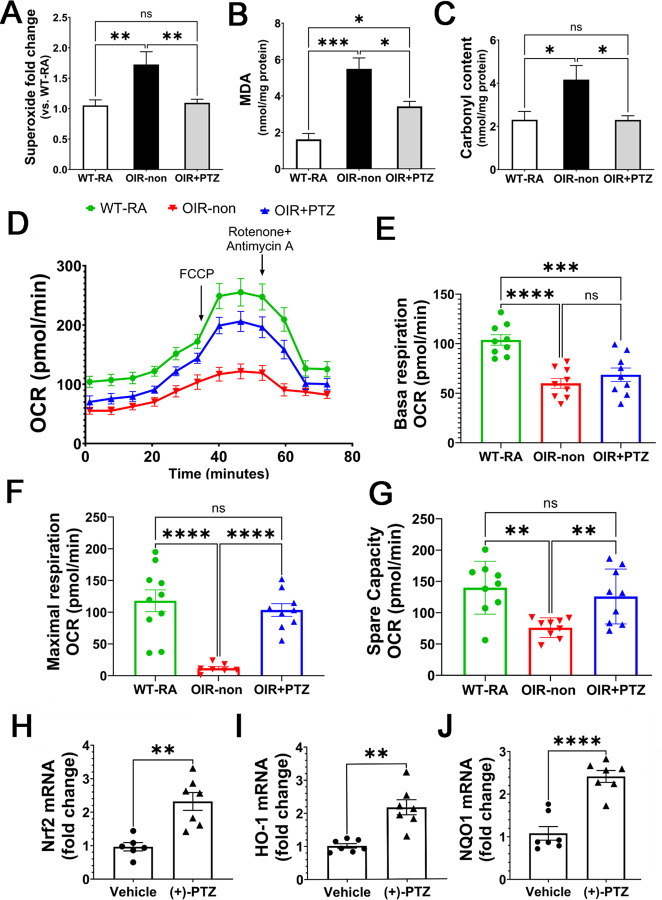
Activation of Sig1R mitigates mitochondrial dysfunction and enhances Sig1R/Nrf2 signaling in OIR mice. (**A**) Superoxide level, (**B**) MDA level, (**C**) Carbonyl protein level in WT-RA, WT-OIRnon and WT-OIR+PTZ mice. (**D-G**) Seahorse Mito stress assay of retina puncta from WT-RA, WT-OIR-non and WT-OIR+PTZ mice. (**D**) OCR, (**E**) Basal respiration, (**F**) Maximal respiration, and (**G**) Spare Capacity were detected. (**H-J**) qRT-PCR assay of *Nrf2*, *HO1* and *NQO1* gene expression in retina from WT-OIR-non and WT-OIR+PTZ mice. Data are the mean ± SEM of analyses in 9–10 retinas per group. *, *p* < 0.05; **, *p* < 0.01; ***, *p* < 0.001, ****, *p* < 0.0001.

Immunostaining revealed that WT-OIR-non retinas exhibited elevated 3-NT and 4-HNE, indicating pronounced nitrosative stress (Fig.9A) and lipid peroxidation (Fig.9C), whereas WT-OIR+PTZ retinas showed markedly reduced immunoreactivity for both 3-NT and 4-HNE (Fig. 9A–D. Dot blot analysis confirmed these findings, with (+)-PTZ restoring 3-NT and 4-HNE levels toward WT-RA controls (Fig.9E–G). To further assess oxidative stress in OIR retinas, we detected superoxide production and oxidative damage. At P17, OIR-non retinas exhibited significantly elevated superoxide production, lipid peroxidation of MDA, and protein carbonylation compared with WT-RA controls. In contrast, treatment with (+)-PTZ markedly reduced superoxide and oxidative damage to lipids and proteins, restoring these markers toward baseline levels (Fig.10 A–C).

### 2.8. (+)-PTZ mitigates mitochondrial dysfunction and enhances Nrf2 signaling in OIR mice

Mitochondrial respiration was further assessed using a modified seahorse assay in isolated retinas. Basal mitochondrial respiration was significantly reduced in OIR-non retinas compared with WT-RA controls, indicating suppressed oxidative metabolism. Although (+)-PTZ treatment showed a trend towards increased basal respiration, it did not reach statistical significance (Fig.10D, E). In contrast, FCCP-stimulated maximal respiration dramatically decreased in OIR-non retinas (Fig.10 F), resulting in a robust loss of spare respiratory capacity (Fig.10 G). However, both maximal respiration and spare capacity were significantly restored in OIR+PTZ retinas to levels comparable to WT-RA retinas (Fig. D-G). Sig1R localizes on MAM, where it has been implicated in the regulation of mitochondrial function and redox homeostasis. Given the established involvement of Nrf2 in Sig1R-mediated redox regulation, we next examined Nrf2 signaling. qRT-PCR analysis demonstrated that *Nrf2* mRNA levels were significantly increased in OIR+PTZ retinas compared with OIR-non retinas (Fig.10H). Moreover, the expression of two key Nrf2 downstream antioxidant genes, HO-1 (Fig.10I) and NQO1 (Fig.10J), were significantly upregulated following (+)-PTZ treatment.

## 3. Discussion

This study demonstrates that pharmacological activation of Sig1R provides sustained neuroprotection in experimental ROP. Long-term treatment with the high-affinity Sig1R agonist (+)-PTZ preserved visual acuity, contrast sensitivity, rod pathway function, RGC activity, retinal structure, and neuronal survival following OIR. Importantly, these protective effects were completely abolished in Sig1R-deficient mice, establishing a direct requirement for Sig1R signaling. Together, these findings identify Sig1R as a critical regulator of retinal neurovascular unit integrity and support Sig1R activation as a potential disease-modifying strategy for ROP.

Although current ROP therapies effectively suppress pathological NV, they do not directly address the neuronal dysfunction that contributes to long-term visual impairment. In the present study, Sig1R activation restored visual function toward WT-RA levels while preserving retinal thickness and RGC survival (Fig.2–5). When considered together with our recent findings that (+)-PTZ reduces pathological NV and vascular leakage in OIR (Wang 2026), our data support a coordinated neurovascular protective role for Sig1R activation. These findings suggest that targeting cellular stress responses may complement existing vascular-directed therapies by preserving overall retinal integrity and visual function.

One strength of this study is the genetic validation of Sig1R dependence. Although (+)-PTZ is a highly selective Sig1R agonist, pharmacological studies alone cannot fully exclude off-target effects. Here, the complete loss of protection in Sig1R^−/−^ OIR mice demonstrates that preservation of visual function, retinal structure, and neuronal survival requires Sig1R signaling. These findings are consistent with previous studies in retinal degenerative disease model (Wang, Saul, et al. 2016), establishing Sig1R as a critical mediator of the protection observed in OIR.

Mechanistically, our data supports a hypothesis that Sig1R functions as an upstream regulator of retina stress adaptation. Oxidative stress, mitochondrial dysfunction, and apoptosis are widely recognized drivers of retinal injury in ROP and other ischemic retinal diseases. Consistent with this, WT-OIR-non retinas exhibited marked oxidative and nitrosative damage accompanied by impaired mitochondrial respiration (Fig. 9, 10). Sig1R activation markedly suppressed oxidative injury, improved maximal respiration and spare respiratory capacity, and enhanced expression of Nrf2 and its downstream antioxidant targets HO1 and NQO1 (Fig.10). These findings are consistent with the established localization of Sig1R at MAM where it regulates calcium signaling, mitochondrial homeostasis, and cellular stress responses (Schmidt et al. 2016; Hayashi and Su 2003). In parallel, (+)-PTZ reduced Bim expression, cytochrome c release, and caspase-9 activation, indicating suppression of apoptotic signaling (Fig.8). Collectively, these results suggest that Sig1R activation stabilizes mitochondrial function, enhances endogenous antioxidant defenses, and limits stress-induced apoptosis, thereby promoting long-term neuronal survival after ischemic retinal injury.

An important question addressed by this study was whether Sig1R activation provides durable protection rather than simply delaying degeneration. Our longitudinal analyses extending to 20 weeks demonstrated sustained preservation of visual function, retinal structure, and neuronal survival, supporting a true neuroprotective effect. Because visual deficits frequently persist after regression of acute vascular pathology in ROP, the long-term protection observed here suggests that Sig1R activation targets fundamental mechanisms contributing to chronic retinal dysfunction.

The cellular basis of Sig1R-mediated protection remains to be defined. Sig1R is expressed in multiple retinal cell populations, including neurons, Müller glia, microglia, retinal pigment epithelial cells (Smith et al. 2018; Wang, Cui, et al. 2017; Wang et al. 2015; Ola et al. 2001; Shanmugam et al. 2016), raising the possibility that the observed protection reflects coordinated multicellular responses within the retinal neurovascular unit. While previous studies support direct neuronal protection by Sig1R activation (Shanmugam et al. 2015; Wang et al. 2015), the relative contributions of specific retinal cell type were not resolved in the present study. Future investigations using conditional Sig1R knockout models via our recently developed Sig1R flox/flox mice (Huang et al. 2022), will help define the cellspecific mechanisms responsible for neurovascular protection.

Several limitations should also be considered. Although OIR is the most widely used experimental model of ROP, it does not fully recapitulate the complexity of human disease. In addition, the optimal dosing regimen, therapeutic window, and developmental safety of chronic Sig1R activation remain to be established. Future studies evaluating delayed treatment paradigms and combination approaches with current anti-VEGF therapies will be important for clinical translation.

Despite these limitations, the therapeutic promise of targeting Sig1R is supported by substantial evidence across multiple neurodegenerative and neurovascular disorders. Sig1R activation has demonstrated protective effects in models of retinal degeneration, diabetic retinopathy, glaucoma, ischemic injury (Wang, Saul, et al. 2016; Wang, Cui, et al. 2016; Zhao et al. 2017; Wang et al. 2021; Wang et al. 2020; Xiao et al. 2020; Wang and Smith 2019; Wang, Saul, and Smith 2019; Smith et al. 2018; Wang, Saul, et al. 2017; Wang, Cui, et al. 2017), and several CNS degenerative diseases (Ryskamp et al. 2017; Benarroch 2018; Arnold 2021). Importantly, clinically relevant Sig1R agonists are already undergoing evaluation in human neurological disorders. Pridopidine, a high-affinity Sig1R agonist, has advanced into clinical studies for Huntington disease (Ryskamp et al. 2017) and amyotrophic lateral sclerosis (Benarroch 2018), while fluvoxamine has demonstrated Sig1R-dependent anti-inflammatory activities in clinical trials (Lenze, Reiersen, and Santosh 2022; Bhuta et al. 2022). The ongoing clinical development of Sig1R-targeted therapies provide a significant translational advantage and support the feasibility of modulating this pathway in human disease.

In summary, Sig1R activation preserves long-term visual function, retinal structure, and neuronal survival in experimental ROP. The protective effects are associated with reduced oxidative and nitrosative stress, improved mitochondrial bioenergetics, enhanced Nrf2 antioxidant signaling, and suppression of apoptosis, which are abolished in Sig1R-deficient mice. Together, these findings identify Sig1R as a central regulator of retinal stress and support Sig1R-targeted therapies as a promising strategy for preserving neurovascular unit integrity in ROP, as well as retinal and CNS neurovascular diseases characterized by hypoxic and oxidative stress.

## 4. Methods

### 4.1. Animals

Male and female C57BL/6J wildtype (WT) and Sigma 1 receptor knockout (Sig1R^−/−^) mice (Sabino et al. 2009; Wang et al. 2015), both on C57BL/6J background, were maintained under standard conditions (12-h light/dark cycle, Teklad 2918 diet). All procedures complied with institutional guidelines, the ARVO statement for the use of Animals in Ophthalmic and Vision Research, and the ARRIVE guidelines.

### 4.2. Oxygen induced retinopathy (OIR) mouse model

OIR was induced as described by Smith et al. with minor modifications (Smith et al. 1994; Connor et al. 2009). On postnatal day 7 (P7), pups with their dams were exposed to 70% oxygen for 5 days and then returned to room air (RA; 21% oxygen) until analysis. RA-reared littermates served as controls. Data were pooled across litters to minimize variability due to differences in litter size and weight (Kim, D’Amore, and Connor 2016). Data from males and females were combined, as no sex-related differences were observed in OIR model as suggested before (Smith et al. 1994).

### 4.3. (+)-Pentazocine [(+)-PTZ] treatment

The dextrorotatory chiral enantiomer of pentazocine, (+)-PTZ, was administered via intraperitoneal injection *(i.p.)* at a dose of 0.5 mg/kg body weight (Sigma-Aldrich, P127, St. Louis, MO, USA) according to our previous studies (Wang, Saul, et al. 2016). Treatment began on P7 at the onset of hyperoxia exposure and continued every other day until the experimental endpoint (Wang, Saul, and Smith 2019).

### 4.4. Visual acuity and contrast sensitivity

Visual acuity and contrast sensitivity were assessed using the OptoMotry (OMR) system (Cerebra Mechanics, Medicine Hat, Alberta, Canada) as previously described (Barwick et al. 2023; Guo et al. 2023; Prusky et al. 2004). Briefly, unrestrained mice were placed on a center pedestal with a chamber of four monitors displaying vertical sine-wave gratings moving at 12°/s. Visual acuity (spatial frequency threshold) was determined by increasing grating frequency at 100% contrast until tracking ceased using a method of limits yes/no criterion. Contrast threshold (%) was measured at 0.092 cycles/degree by determining contrast from 100% until detection failed. Contrast sensitivity (CS) was calculated as the reciprocal of the contrast threshold (CS=1/contrast threshold).

### 4.5. ERG and PERG

ERG recordings were performed using the Celeris Ophthalmic Electrophysiology System (Diagnosys, owell, MA) following established protocols (Wang et al. 2021; Wang, Xu, and Caldwell 2025). Anesthetized mice with dilated pupils (0.5% tropicamide and 2.5% phenylephrine) were placed on a heated platform (37°C) with corneal hydration maintained with 0.3% Hypromellose gel. Scotopic ERGs were recorded after overnight dark adaptation using flash intensities of 0.001, 0.005, 0.01, 0.1, 0.5, and 1.0 cd s/m^2^. Photopic ERGs were recorded after light adaptation with rod saturation, using intensities of 3, 10, 25, 50, 100, and 150 cd s/m^2^.

PERG was performed to assess retinal ganglion cell (RGC) function in dark-adapted mice(Xiao et al. 2022). The pattern stimulator contacted the tested eye, with the contralateral flash electrode as reference. Transient responses were elicited by alternating black-white horizontal bars (0.155 cycles/degree, 50 cd s/m^2^, 100% contrast) and averaging over 600 sweeps to optimize signal quality.

### 4.6. Spectral Domain-Optical Coherence Tomography (SD-OCT)

SD-OCT was used to evaluate retinal structure *in vivo*. Under anesthesia, retinal images were acquired with a Bioptigen SD-OCT Imaging System (Leica, R2200, Buffalo Grove, IL). Single B-scan and volumetric intensity scans were centered on the optic nerve as described previously (Wang et al. 2020). Retinal layer segmentation and thickness measurements were performed using InVivoVue^™^ Diver 2.4 software. Parameters assessed included total retinal thickness (TRT), retinal nerve fiber layer (RNFL), inner plexiform layer (IPL), inner nuclear layer (INL), outer plexiform layer (OPL), outer nuclear layer plus inner segments (ONL + IS), outer segments (OS), and retinal pigment epithelium (RPE). Layer thickness of each retina was individually plotted, and data for a given retinal layer was averaged for each group.

### 4.7. JB4 processing microscopic analysis

Mice were euthanized and eyes were collected for JB-4 methacrylate embedding (Electron Microscopy Sciences). For plastic embedding, eyes were immersion-fixed in 2% paraformaldehyde/2% glutaraldehyde in 0.1 M cacodylate buffer before JB-4 processing. Sections were stained with hematoxylin and eosin (H&E), imaged using an Axioplan-2 microscope equipped with a high-resolution camera and analyzed with Zeiss Axiovision software (version 4.7). Plastic-embedded retinal sections were examined for gross structural changes and evaluated morphometrically. Measurements included TRT, the thickness of the INL, ONL, IPL, and OPL, the length of inner/outer segments, and the number of RGC. Six images per retina (temporal and nasal to the optic nerve) were analyzed to capture data across central, midperipheral, and peripheral retinal regions.

### 4.7. Retinal cryosection immunostaining

Retinal cryosections (10 μm) were prepared from snap-frozen eyeballs. Cryo-sectioned slides were fixed in 4% paraformaldehyde and incubated overnight at 4°C with the anti-4-hydroxynonenal (4-HNE; 1:100; Abcam, Cambridge, MA, USA), anti–3-nitrotyrosine (3-NT; 1:1000; Cayman Chemical, Ann Arbor, MI, USA) antibodies. Slides were then washed three times with 0.1% Triton X-100 in 0.1 M phosphate-buffered saline (PBS; pH 7.4) and incubated with appropriate fluorescence-conjugated secondary antibodies (Invitrogen, Thermo fisher). Sections were mounted with Fluor shield mounting medium containing DAPI (Sigma-Aldrich, St. Louis, MO, USA) and imaged at 20X magnification using a Zeiss Axio Imager D2 fluorescence microscope (Carl Zeiss, Thornwood, NY, USA).

### 4.8. Detection of oxidative damage

*Lipid peroxidation* was assessed using the thiobarbituric acid reactive substances (TBARS) assay kit (Cayman Chemical). This assay quantifies malondialdehyde (MDA), a byproduct of lipid peroxidation. Absorbance was measured at 532 nm, and MDA concentrations were calculated from a standard calibration curve. *Protein oxidation* was evaluated in retinal lysates using a Protein Carbonyl ELISA kit (Cell Biolabs). Lysates corresponding to two retinas per assay were applied to the 96-well protein-binding plates provided with the kit. Samples were derived with 2,4-dinitrophenylhydrazine (DNPH) and incubated sequentially with an anti-dinitrophenyl antibody and an HRP-conjugated secondary antibody. Absorbance was measured at 450 nm using a microplate reader (Molecular Devices). Protein carbonyl content was calculated using the bovine serum albumin standard supplied with the kit. *Superoxide anion levels* were quantified using a superoxide anion assay kit (CS1000, Sigma-Aldrich). Retinas were dissected and incubated in 200μl Krebs/HEPES buffer at 37°C under 5% CO_2_ in the dark for 10 min. Xanthine working solution was added to each well to initiate the reaction. Luminescence intensity was measured for 3–5 minutes at 30s intervals. Measurements were repeated over a 30 min period. After averaging and subtracting blank values, results were normalized to protein concentration.

### 4.9. TUNEL assay

Cell death was assessed in retinal cryosections using the ApopTag Fluorescein Direct in Situ Apoptosis Detection Kit (Millipore). Sections were counterstained with DAPI and imaged via Zeiss Axio Imager D2. For each mouse, at least five retinal sections were analyzed, with six images (three temporal and three nasal) captured per retina. TUNEL-positive cells were quantified by masked observers, and results were expressed as the number of TUNEL-positive cells per square millimeter of retinal area.

### 4.10. Mitochondrial respiration

Mice were anesthetized with ketamine/xylazine, and retinas were rapidly dissected into Hank’s balanced salt solution (HBSS). Middle retinal punches (1 mm) were obtained under a dissecting microscope using biopsy punches and placed into a Seahorse XF24 islet capture microplate (one punch per well), oriented ganglion cell layer up. Plate was incubated at 37 °C in a non-CO_2_ incubator for 45–60 min prior to the assay. Oxygen consumption rate (OCR) was evaluated using the Agilent Seahorse XF Cell Mito Stress Test kit (*Cat. No*. 103015–100) according to the manufacturer’s instructions. Seahorse Assay Medium (Seahorse XF DMEM, Ph 7.4) was supplemented with 2 mM glutamine, 0.5 mM sodium pyruvate and 12 mM glucose. Carbonyl cyanide-4 (trifluoromethoxy) phenylhydrazone (FCCP) and rotenone/antimycin A (Rot/AA) were serially injected to final concentration of 0.5 μM to measure basal respiration, maximal respiration and spare capacity. Data were analyzed with Wave software (Agilent, Santa Clara, CA, United States) and normalized to protein content.

### 4.11. Dot/Western blot

**For dot blot:** Equal amounts of protein from whole retinal lysates were spotted onto nitrocellulose membranes and air dried for 5 min at room temperature. Membranes were blocked for 1 h with 5% skim milk in PBS, then incubated for 1 h with either anti-3-nitrotyrosine (3-NT; 1:1000, Cayman, Ann Arbor, MI, USA) or anti-4-HNE (1:1000, Abcam, Cambridge, MA, USA) antibodies in PBS-tween buffer. After three washes in PBS-tween, membranes were incubated with horseradish peroxidase-conjugate secondary antibody (1:5,000; Cell Signaling, MA, USA). Immuno-positive spots were visualized using Clarity ECL blotting substrate (Bio-Rad, Hercules, CA, USA). **For western blot:** Proteins were extracted from neural retina and separated by SDS-PAGE. Following transfer to nitrocellulose membranes, blots were incubated with primary antibodies as following: anti-Bim (1:1000, Cell Signaling Tech, 2933S), anti-cytochrome C (1:1000, Cell Signaling Tech, 4272S), anti-cleaved caspase 9 (1:1000, Cell Signaling Tech, 9507S) and tubulin (1:2000, Sigma-Aldrich, T5326). After secondary antibody incubation, the membrane was visualized using the Super Signal West Pico Chemiluminescent Substrate detection system (Pierce Biotechnology). Band intensities were quantified using ImageJ 1.48v software.

### 4.12. qRT-PCR

Total RNA was isolated from neural retinas using TRIzol reagent (Invitrogen) according to the manufacturer’s instructions and quantified. Two micrograms of total RNA were reverse transcribed using the iScript cDNA Synthesis Kit (Bio-Rad Laboratories, Hercules, CA, USA). Quantitative PCR was performed using SsoAdvanced SYBR Green Supermix (Bio-Rad Laboratories) and gene-specific primers on a CFX96 Touch Real-Time PCR Detection System (Bio-Rad Laboratories) with 40 amplification cycles. The following primers were used: *Nrf2*-Forward: 5’-*TAGATGACCATGAGTCGCTTGC*-3’, Reverse: 5’-*GCCAAACTTGCTCCATGTCC*-3’; *HO-1*-Forward: 5’-*AAGCCGAGAATGCTGAGTTCA*-3’, Reverse: 5’*GCCGTGTAGATGTGGTACAAGGA*-3’; *NQO1*-Forward: 5’-*AGGATGGGAGGTACTCGAATC*-3’, Reverse: 5’-*AGGCGTCCTTCCTTATATGCTA*-3’. Relative gene expression levels were calculated using the comparative ΔΔCt method.

### 4.13. Data analysis

All values are presented as mean ± *SEM*. One-way or two-way ANOVA with Tukey post-hoc testing was performed using GraphPad Prism 10 software (GraphPad, La Jolla, CA). Significance was defined as *p* < 0.05.

## Highlights:

- Sig1R activation preserves long-term visual function after neonatal retinal injury.
- (+)-Pentazocine protects retinal ganglion cell and rod pathway function.
- Sig1R-dependent neuroprotection is confirmed using Sig1R knockout mice.
- Sig1R activation improves mitochondrial function and antioxidant signaling.
- Targeting Sig1R promotes coordinated neurovascular protection in OIR.
